# Different Pathophysiology and Outcomes of Heart Failure With Preserved Ejection Fraction Stratified by K-Means Clustering

**DOI:** 10.3389/fcvm.2020.607760

**Published:** 2020-11-30

**Authors:** Daisuke Harada, Hidetsugu Asanoi, Takahisa Noto, Junya Takagawa

**Affiliations:** ^1^The Cardiology Division, Imizu Municipal Hospital, Toyama, Japan; ^2^Toyama Nishi General Hospital, Toyama, Japan

**Keywords:** heart failure with preserved ejection fraction, artificial intelligence, stratified medicine, machine learning, K-means clustering, right ventricular distensibility, systolic pulmonary arterial pressure (SPAP), cardio renal syndrome

## Abstract

**Background:** Stratified medicine may enable the development of effective treatments for particular groups of patients with heart failure with preserved ejection fraction (HFpEF); however, the heterogeneity of this syndrome makes it difficult to group patients together by common disease features. The aim of the present study was to find new subgroups of HFpEF using machine learning.

**Methods:** K-means clustering was used to stratify patients with HFpEF. We retrospectively enrolled 350 outpatients with HFpEF. Their clinical characteristics, blood sample test results and hemodynamic parameters assessed by echocardiography, electrocardiography and jugular venous pulse, and clinical outcomes were applied to k-means clustering. The optimal k was detected using Hartigan's rule.

**Results:** HFpEF was stratified into four groups. The characteristic feature in group 1 was left ventricular relaxation abnormality. Compared with group 1, patients in groups 2, 3, and 4 had a high mean mitral E/e′ ratio. The estimated glomerular filtration rate was lower in group 2 than in group 3 (median 51 ml/min/1.73 m^2^ vs. 63 ml/min/1.73 m^2^ *p* < 0.05). The prevalence of less-distensible right ventricle and atrial fibrillation was higher, and the deceleration time of mitral inflow was shorter in group 3 than in group 2 (93 vs. 22% *p* < 0.05, 95 vs. 1% *p* < 0.05, and median 167 vs. 223 ms *p* < 0.05, respectively). Group 4 was characterized by older age (median 85 years) and had a high systolic pulmonary arterial pressure (median 37 mmHg), less-distensible right ventricle (89%) and renal dysfunction (median 54 ml/min/1.73 m^2^). Compared with group 1, group 4 exhibited the highest risk of the cardiac events (hazard ratio [HR]: 19; 95% confidence interval [CI] 8.9–41); group 2 and 3 demonstrated similar rates of cardiac events (group 2 HR: 5.1; 95% CI 2.2–12; group 3 HR: 3.7; 95%CI, 1.3–10). The event-free rates were the lowest in group 4 (*p* for trend < 0.001).

**Conclusions:** K-means clustering divided HFpEF into 4 groups. Older patients with HFpEF may suffer from complication of RV afterload mismatch and renal dysfunction. Our study may be useful for stratified medicine for HFpEF.

## Introduction

The rate of heart failure with preserved ejection fraction (HFpEF) increases with age, reaching 50% or higher in patients with heart failure (1). Many previous studies revealed that HFpEF has many aspects, and the heterogeneity of this syndrome suggests different etiological and pathophysiological paths by which individual patients develop heart failure (2–5). This heterogeneity also impedes the effectiveness of existing medications, such as inhibitors of the renin-angiotensin system and/or beta blockers for heart failure with reduced ejection fraction, and is related to poor outcomes for patients with HFpEF (6, 7). Thus, the one-size-fits-all approach cannot improve clinical outcomes and precision medicine may be needed for patients with HFpEF (8). Although the individual pathophysiology must be known to perform precision medicine, common pathophysiologies for HFpEF may exist. By identifying subgroups of patients with different pathophysiologies of HFpEF, stratified medicine may enable the development of effective treatments for particular groups of patients with HFpEF; however, the multidimensionality of HFpEF makes it difficult to group patients together by common disease features. To overcome this problem, the precise calculating ability of artificial intelligence helped to stratify HFpEF. Indeed, using several machine-learning algorithms, previous studies clarified the phenotypes and therapeutic strategies for HFpEF; however, the features of heart failure with mid-range ejection fraction may influence the features of unknown phenotypes and RV diastolic function was not taught in previous studies (9–13). Although RV function plays an important role in the pathophysiology of HFpEF (14), there is a lack of guidance for the assessment and quantification of RV diastolic function (15). The physiological properties of the right ventricle are lower contractility and higher compliance than the left ventricle (16). The loss of high compliance, the greatest feature of the right ventricle, will influence on clinical outcomes of HFpEF. Indeed, we reported that the rate of less-distensible right ventricle assessed by jugular venous pulse increased with age and was risk factor for cardiac events of HFpEF (17, 18). If this feature is taught in machine learning, a new important subgroup may be found. By enrolling patients meeting the diagnostic criteria of HFpEF described in heart failure guideline (19) and teaching cardiac function by referring to echocardiographic and jugular venous pulse evaluation, this study aimed to clarify new subgroups of HFpEF using machine learning.

## Materials and Methods

**Figure 1:**
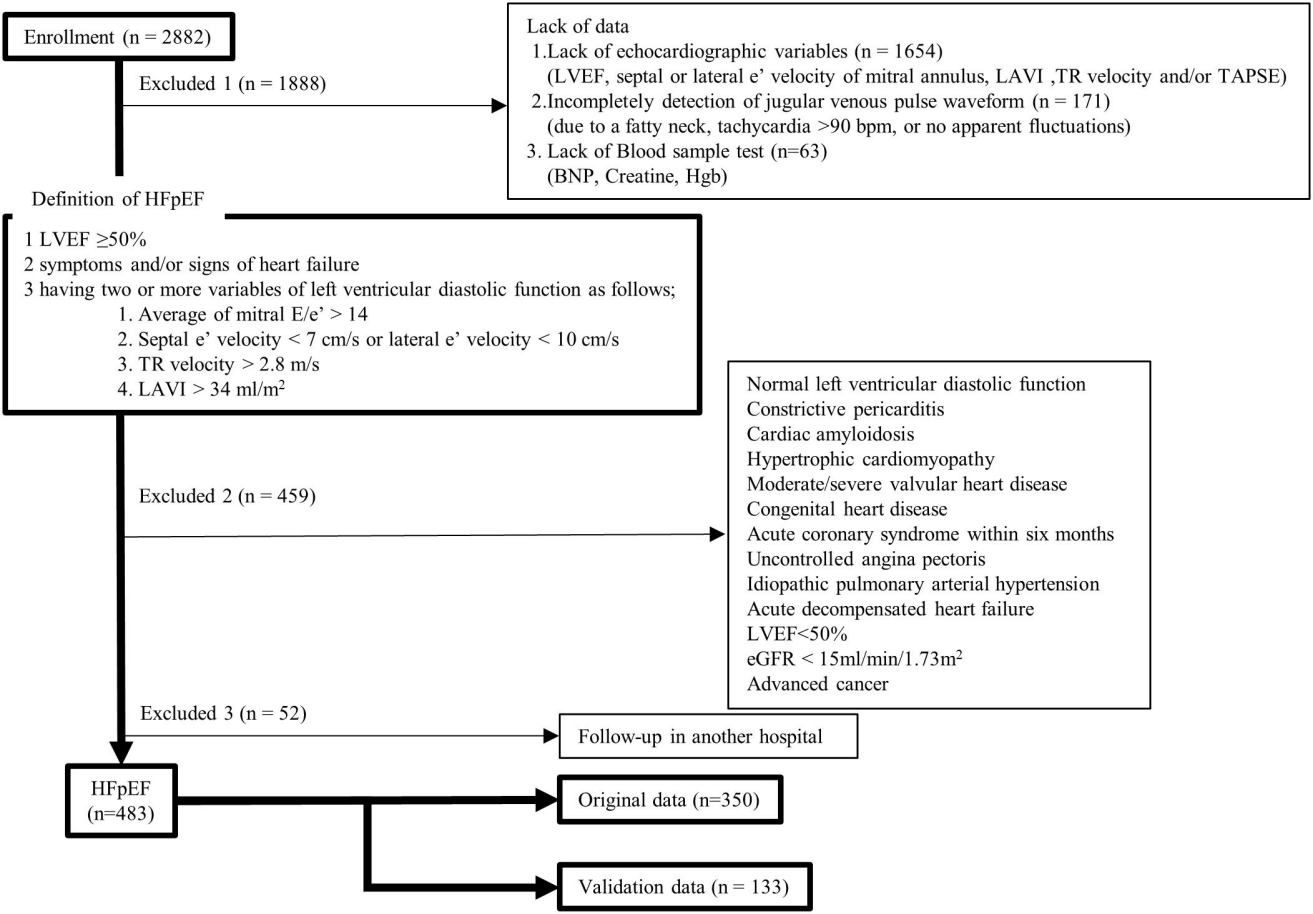
Study flowchart. BNP, brain natriuretic peptide; eGFR, estimated glomerular filtration rate; HFpEF, heart failure with preserved ejection fraction; Hgb, hemoglobin; LAVI, left atrial volume index; LVEF, left ventricular ejection fraction; TAPSE, tricuspid annular plane systolic excursion; TR, tricuspid regurgitant. [adapted from Figure 1 in (20)].

In this study, after receiving approval from the Human Subject Review Committee of our institute, all data from our echocardiographic and jugular venous pulse database and medical records were retrospectively obtained. Between April 2013 and March 2020, 7,437 consecutive outpatients underwent echocardiographic examinations (Vivid 7, General Electric Healthcare, Wauwatosa, WI, USA) for cardiovascular disease. For 2,882 patients, we simultaneously recorded electrocardiography, phonocardiography, and jugular venous pulse measurement, and all data were stored using a hard-disk memory system (echoPAC PC, General Electric Healthcare) for later analyses. A flowchart of this study is shown in Figure 1. In the present study, we defined patients with HFpEF as those with left ventricular (LV) ejection fraction ≥50%, two or more positive variables of LV diastolic dysfunction, having symptoms and/or signs of heart failure, and a brain natriuretic peptide (BNP) level >35 pg/ml (19, 21). First, patients were excluded if they lacked data, such as LV ejection fraction, mitral e′, left atrial volume index, tricuspid regurgitant velocity, tricuspid annular plane systolic excursion (TAPSE), jugular venous pulse waveform, BNP, creatine, and hemoglobin. Patients were also excluded if they had normal LV diastolic function, constrictive pericarditis, cardiac amyloidosis, hypertrophic cardiomyopathy, moderate or severe valvular heart disease, congenital heart disease, acute coronary syndrome within 6 months, uncontrolled angina pectoris, idiopathic pulmonary arterial hypertension, acute decompensated heart failure, LV ejection fraction <50%, kidney failure (estimated glomerular filtration rate [eGFR] <15 ml/min/1.73 m^2^), or advanced cancer. We diagnosed 535 patients with HFpEF, but 52 were excluded because of follow-up at another hospital. In total, we retrospectively enrolled 483 patients in the present study. The data from 350 patients obtained during the period from April 2015 to March 2020 were used as original data to find new phenotypes of HFpEF and data from other 133 patients obtained during the period from April 2013 to March 2015 were used to validate the phenotypes found by clustering methods. All patients took medications continuously for 4 months. Based on the arrangement of our hospital, informed consent was provided by all patients at the time when they were examined using echocardiography and/or underwent blood sample tests. The study complied with the Declaration of Helsinki.

### Evaluation of Cardiac Function

Cardiac function was evaluated as in our previous report (17, 18, 20). The jugular venous pulse waveform was used to evaluate RV distensibility. It was recorded in the supine position by well-trained cardiac sonographers. A pulse-wave transducer (TY-306, Fukuda Denshi, Tokyo, Japan) was placed over the neck, above and to the right of the junction of the right clavicle and the manubrium sterni, and held in place manually. The jugular venous waveform was recorded for at least 30 s and digitized at a sampling interval of 600 Hz. Using an off-line moving average technique (Matlab version 14, Mathworks, Natick, MA, USA), respiratory baseline fluctuations (0.1–0.5 Hz) were excluded from the jugular waveform to determine the relative depth of the nadirs of “X” and “Y” descent (17, 18, 20). According to the established significance of the jugular venous waveform (22–24), two cardiologists who were blinded to the clinical data judged whether the jugular venous pulse had a dominant “Y” descent, where the nadir of the “Y” descent was deeper than that of the “X” descent, reflecting a less-distensible right ventricle. LV end-diastolic and end-systolic volumes were measured using a modification of Simpson's method. The LV ejection fraction was calculated as stroke volume divided by end-diastolic volume. LV mass was also calculated using the Devereux formula and was divided by surface area (LV mass index [LVMI]) (25). To evaluate the diastolic properties of the left ventricle, we measured the early diastolic velocities (e′) using pulsed-wave tissue Doppler from the apical view. We measured the septal and lateral E/e′, and averaged the values for more reliable assessment of LV relaxation and filling pressure (21). If patients had atrial fibrillation (AF), we estimated velocity measurements from 10 consecutive cardiac cycles (21). The left atrial volume index was obtained using the biplane method from both the apical four- and two-chamber views (25). In addition, tricuspid regurgitant jet was detected using the continuous Doppler technique to measure the RV systolic pressure. The peak pressure gradient from the right ventricle to the right atrium was calculated from the peak tricuspid regurgitant velocity (V) using a modified Bernoulli equation (pressure gradient = 4 V^2^). The peak RV pressure was then calculated by adding the peak pressure gradient to the right atrial pressure, which was estimated from the echocardiographic characteristics of the inferior vena cava (26). We regarded RV systolic pressure as systolic pulmonary arterial pressure (SPAP) because of the absence of a gradient of across the pulmonic valve and RV outflow tract. The LV ejection fraction, mean mitral e′, mean mitral E/e′ ratio, SPAP, TAPSE, and jugular venous pulse waveform were used as indicators of LV contractility, LV relaxation ability, LV filling pressure, RV afterload, RV contractility, and RV diastolic function, respectively, in this study.

### K-Means Clustering

We used R and downloaded several packages to perform k-means clustering to stratify patients with HFpEF (27–29). K-means clustering, unsupervised machine learning, is one of the most popular clustering techniques. K-means clustering produces hard (an element can only be a member of one cluster), flat, and polythetic (membership is determined by similarity based on multiple attributes) clusters. The k-means algorithm has no training or testing data *per se*. It works by creating each cluster around a centroid, which is an average cluster member, namely, the center of a cluster (30). The steps of the k-means clustering algorithm are as follows: First, the algorithm starts by specifying the number of clusters (k). Second, k random centroids are initialized based on datapoints in the data. Third, for each point, the algorithm finds the nearest centroid and assigns the point to that cluster. To find the nearest centroid, Euclidean distance was used in this study. Fourth, the centroid is adjusted such that it minimizes the distance within the cluster variance. Lastly, the algorithm stops once cluster assignment stops making changes (30). It is well-known that the number of clusters specified greatly affects the performance of k-means clustering. To determine the optimal k, Hartigan's rule was used in this study. The Euclidean distance formula is not defined for nominal data. To calculate the distance between nominal features, they need to be converted into a numeric format, for which we used dummy coding, where a value of one indicates one category, and zero, indicates the other (31, 32). To avoid some features having a larger range of values than the others solely dominating, the features applied for k-means clustering were standardized using z-scores, as in the following formula (31):

(1) $$\begin{array}{r} Z-score=\left(x-\mu \right)/\sigma \end{array}$$

where μ is the mean of x and σ is the standard deviation of x. When the values were standardized by z-scores, positive values were above the overall mean level and negative values were below the overall mean. By examining whether the clusters fall above or below the mean level for each interest category, we can begin to identify patterns that distinguish the clusters from each other. An extreme z-score reflects the features of the cluster (31). Principal component analysis was also applied to visualize the results of k-means clustering (33).

### Documentation of End Points

All 483 patients were followed up at the outpatient clinic of our hospital. We defined deterioration of HFpEF as follows: sudden death, death from heart failure, or hospitalization for deterioration of HFpEF. These cardiac events were reported and adjudicated by cardiovascular specialists at our hospital.

### Validation Cohort

We performed an independent validation analysis on 133 of 458 patients (the data obtained in the period from April 2013 to March 2015). Setting the same number as clusters estimated from original cohort, k-means clustering was also performed in the validation cohort. We then looked to see whether there was again a difference in outcomes among the groups using same outcome analysis (cox proportional hazards analysis) used in the original cohort.

### Statistical Analysis

Numerical data are expressed as the median (interquartile range), mean ± standard deviation, or z-score. The Shapiro–Wilk test was used to assess the normality of data. To assess homogeneity of variance, Bartlett's test was used in this study. One-way analysis of variance or the Kruskal–Wallis test was used to compare numerical data among groups, and the chi-square test or Fisher's exact test was used to compare non-parametric data among groups. If a significant difference was observed among groups, Holm's method was used to compare the groups. For outcomes analyses, we used unadjusted and age-adjusted Cox proportional hazards models to determine the independent association between groups and outcomes. Cardiac events of HFpEF stratified by k-means clustering were estimated using the Kaplan–Meier method. Differences between the event-free curves were examined using the log-rank chi-square test and Holm's method. Significance was established at *p* < 0.05. All statistical analyses were carried out using EZR (Saitama Medical Center, Jichi Medical University, Saitama, Japan) (34).

## Results

**Figure 2:**
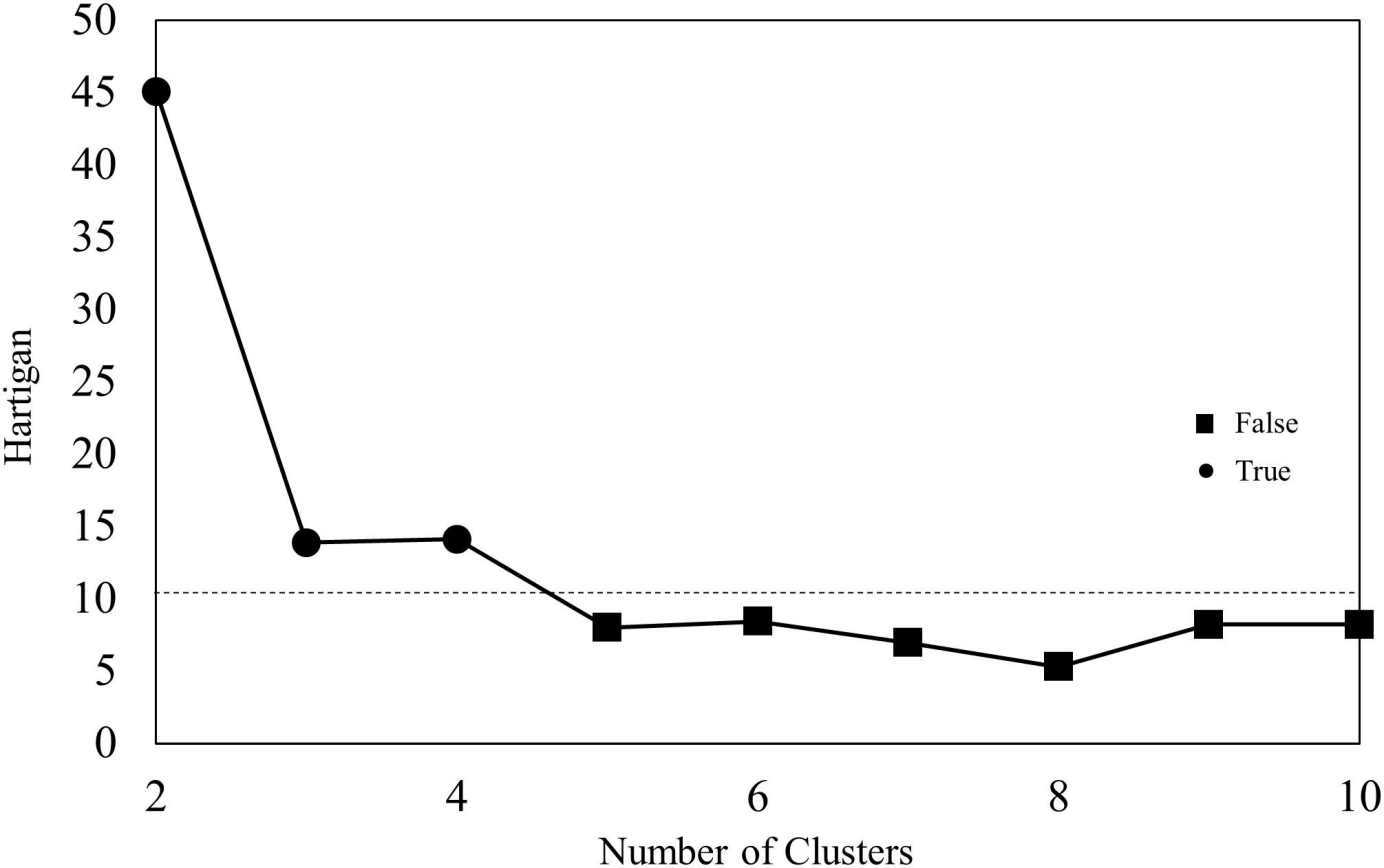
Results of Hartigan's rule.

**Figure 3:**
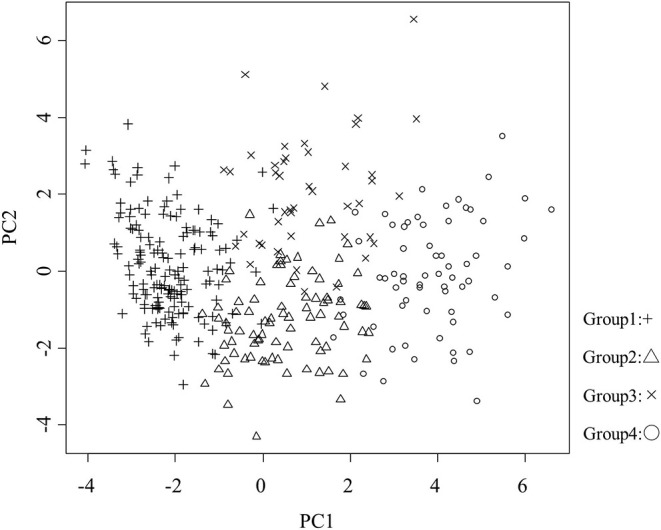
Visualization of the results of k-means clustering. Proportions of variance of principle components 1 and 2 were 15.6 and 6.8%, respectively. PC, principle component; +, group 1; △, group 2; ×, group 3; ○, group 4.

Patient characteristics, renal function, hemoglobin level, cardiac function, and the rate of cardiovascular events are shown in Table 1. These 37 features were applied to k-means clustering in this study. The optimal k was four, which was detected using Hartigan's rule (Figure 2). HFpEF was stratified into four groups by k-means clustering. The coordinates of the cluster centroids according to stratification using k-means clustering are shown in Table 2. Using principle component analysis, the results of k-means clustering are visualized in Figure 3. Coefficients of each feature to create the axes of principle components 1 and 2 are shown in Supplementary Table 1.

**Table 1:** Patients' characteristics.

	**Patients with HFpEF (*n* = 350)**
Age, years	77 (69–83)
Male	159 (45)
BMI, kg/m^2^	24.0 ± 3.7
Heart rate, bpm	68 (62–76)
Systolic blood pressure, mmHg	121 (109–133)
Diastolic blood pressure, mmHg	69 (59–82)
Mean blood pressure, mmHg	87 (79–95)
Underlying disorders	
Hypertension	310 (89)
Diabetes mellitus	71 (20)
Hyperlipidemia	116 (33)
COPD	34 (10)
Prior coronary revascularization	86 (25)
Atrial fibrillation	82 (23)
Medications	
ACEI/ARB	250 (71)
Beta-blockers	187 (53)
Calcium channel blockers	188 (54)
Loop diuretics	158 (45)
eGFR, ml/min/1.73 m^2^	62 (49–76)
Hemoglobin, g/dl	12.5 (11.1–13.6)
Symptoms and signs of HFpEF	
Dyspnea on exertion	337 (96)
Leg edema	144 (41)
Neck vein dilatation	87 (25)
Pleural effusion	64 (18)
Cardiac function	
Left heart	
LAVI, ml/m^2^	38 (34–44)
LVMI, g/m^2^	116 (100–141)
LVEF, %	67 (60–73)
LVEDD, mm	48 (44–52)
DT of mitral inflow	208 (173–241)
Mean mitral e′, cm/s	7.6 (5.8–8.5)
Mean mitral E/e′ ratio	10.6 (8.4–14.2)
Right heart	
RVOT, mm	26 (23–30)
TAPSE, mm	20 (18–23)
SPAP, mmHg	29 (23–36)
Less-distensible right ventricle	135 (39)
Inferior vena cava, mm	13 (11–16)
Cardiac events	80 (23)

*Data are the number of patients (%), median (interquartile range), mean ± SD. ACEI/ARB, angiotensin-converting enzyme inhibitors/angiotensin-receptor blockers; BMI, body mass index; COPD, chronic obstructive pulmonary disease; DT, deceleration time; eGFR, estimated glomerular filtration rate; HFpEF, heart failure with preserved ejection fraction; LAVI, left atrial volume index; LVEDD, left ventricular end-diastolic dimension; LVEF, left ventricular ejection fraction; LVMI, left ventricular mass index; RVOT, right ventricular outflow tract; SPAP, systolic pulmonary arterial pressure; TAPSE, tricuspid annular plane systolic excursion*.

**Table 2:** The coordinates of the cluster centroids.

	**Group 1**	**Group 2**	**Group 3**	**Group 4**
Age	−0.611	0.609	−0.079	0.746
Male	0.226	−0.368	0.183	−0.190
BMI	0.259	−0.238	0.320	−0.538
Heart rate	−0.261	0.089	0.489	0.187
Systolic blood pressure	0.035	−0.050	−0.085	0.038
Diastolic blood pressure	−0.009	−0.073	0.176	−0.000
Mean blood pressure	0.009	−0.096	0.118	0.023
Underlying disorders				
Hypertension	−0.161	0.211	0.145	0.015
Diabetes mellitus	0.066	0.022	−0.278	0.001
Hyperlipidemia	0.351	−0.129	−0.510	−0.338
COPD	−0.091	−0.050	−0.021	0.305
Prior coronary revascularization	0.243	−0.024	−0.517	−0.208
Atrial fibrillation	−0.507	−0.525	1.698	0.774
Medications				
ACEI/ARB	−0.129	0.137	0.079	0.079
Beta-blockers	0.116	−0.175	0.250	−0.225
Calcium channel blockers	−0.042	0.244	−0.165	−0.106
Loop diuretics	−0.701	0.534	0.187	0.881
eGFR	0.437	−0.529	0.056	−0.408
Hemoglobin	0.569	−0.693	0.290	−0.675
Brain natriuretic peptide	−0.478	0.343	−0.070	0.766
Symptoms and signs of HFpEF				
Dyspnea on exertion	0.095	0.134	−0.164	−0.299
Leg edema	−0.628	−0.023	0.641	1.131
Neck vein dilatation	−0.486	−0.303	0.266	1.411
Pleural effusion	−0.456	−0.260	−0.296	1.667
Cardiac function				
Left heart function				
LAVI	−0.382	0.019	0.950	0.258
LVMI	−0.118	0.182	0.027	0.030
LVEF	0.065	0.036	−0.072	−0.157
LVEDD	0.127	−0.122	0.123	−0.233
DT of mitral inflow	0.077	0.436	−0.563	−0.381
Mean mitral e′	0.109	−0.291	0.169	0.003
Mean mitral E/e′ ratio	−0.375	0.287	0.446	0.232
Right heart function				
RVOT	−0.046	−0.248	0.586	0.039
TAPSE	0.456	−0.006	−0.565	−0.724
SPAP	−0.461	0.205	0.317	0.639
Less-distensible right ventricle	−0.556	−0.333	1.120	1.036
Inferior vena cava	−0.337	−0.184	0.632	0.636
Cardiac events	−0.422	0.016	−0.165	1.129

*Data are the z-score. Abbreviations are the same as those in Table 1*.

### Stratification of HFpEF Using K-Means Clustering

#### Patient Characteristics and Comorbidities

Patient characteristics and comorbidities are shown in Table 3. Group 1 was composed of younger individuals (median age 70 years) with relatively higher BMI and rate of prior coronary revascularization (35%), in addition to relatively preserved renal function (median eGFR 69 ml/min/1.73 m^2^). Group 2 was characterized by older age (median age 83 years), the highest proportion of women (73%), and lower eGFR (median 51 ml/min/1.73 m^2^). Group 3 exhibited intermediate age (median age 77), with higher BMI, the highest prevalence of atrial fibrillation (95%), and the lowest prevalence of prior coronary revascularization (2%). Group 4 was characterized by older age (median age 85 years), higher proportion of women (64%), higher prevalence of atrial fibrillation (56%), and lower eGFR (median 54 ml/min/1.73 m^2^). The usage rate of loop diuretics was the highest in group 4.

**Table 3:** Patient characteristics and comorbidities according to stratification using k-means clustering.

	**Group 1 (*n* = 157)**	**Group 2 (*n* = 85)**	**Group 3 (*n* = 44)**	**Group 4 (*n* = 64)**	***p*-Value**
Age, years	70 (63–77)	83 (78–86)*	77 (70–80)^#$^	85 (78–88)^+¶^	<0.001
Male	89 (57)	23 (27)*	24 (55)^$^	23 (36)^+!^	<0.001
BMI, kg/m^2^	25 (23–27)	23 (21–25)*	25 (22–27)^$^	22 (20–24)^+¶^	<0.001
Obesity (BMI ≥ 25 kg/m^2^)	64 (41)	23 (27)	24 (55)^$^	12 (19) ^+¶^	<0.001
Heart rate, bpm	65 (60–74)	68 (62–79)	73 (67–82)^#^	72 (64–79)^+^	<0.001
Systolic blood pressure, mmHg	123 (108–136)	118 (107–132)	122 (109–128)	121 (111–133)	0.852
Diastolic blood pressure, mmHg	70 (57–82)	68 (59–79)	72 (64–83)	69 (60–82)	0.569
Mean blood pressure, mmHg	87 (77–96)	86 (78–92)	89 (83–95)	87 (81–95)	0.548
Underlying disorders					
Hypertension	131 (83)	81 (95)*	41 (93)	57 (89)	0.032
Diabetes mellitus	36 (23)	18 (21)	4 (9)	13 (20)	0.230
Hyperlipidemia	78 (49)	23 (27)*	4 (9)^#^	11 (17)^+^	<0.001
COPD	11 (7)	7 (8)	4 (9)	12 (19)	0.078
Prior coronary revascularization	55 (35)	20 (24)	1 (2)^#$^	10 (16)^+¶^	<0.001
Atrial fibrillation	3 (2)	1 (1)	42 (95)^#$^	36 (56)^+!¶^	<0.001
Medications					
ACEI/ARB	103 (66)	66 (78)	33 (75)	48 (75)	0.180
Beta-blockers	93 (59)	38 (45)	29 (66)	27 (42)	0.013
Calcium channel blockers	81 (52)	56 (66)	20 (45)	31 (48)	0.064
Loop diuretics	16 (10)	61 (72)*	24 (55)^#^	57 (89)^+!¶^	<0.001
eGFR, ml/min/1.73 m^2^	69 (58–83)	51 (37–65)*	63 (50–71)^#$^	54 (37–66)^+¶^	<0.001
KDIGO classification					
G3a–G4 (15–59 ml/min/1.73 m^2^)	46 (29)	56 (66)*	20 (45)	40 (63)^+^	<0.001
G3b–G4 (15–44 ml/min/1.73 m^2^)	7 (4)	36 (42)*	6 (14)^$^	22 (34)^‡+^	<0.001
Hemoglobin, mg/dl	13 ± 1	11 ± 1*	13 ± 2^$^	11 ± 2^+¶^	<0.001

Data are the number of patients (%), median (interquartile range), or mean ± SD. KDIGO, Kidney Disease Improving Global Outcomes. RV, right ventricular; RWT, relative wall thickness. Other abbreviations are the same as those in Table 1. Obesity was defined as a BMI ≥ 25 kg/m^2^ based on the criteria proposed by the Japanese Society for the Study of Obesity. *, comparison between groups 1 and 2, p < 0.05; #, comparison between groups 1 and 3, p < 0.05; +, comparison between groups 1 and 4, p < 0.05; $, comparison between groups 2 and 3, p < 0.05; !, comparison between groups 2 and 4, p < 0.05; ¶, comparison between groups 3 and 4, p < 0.05; ‡, comparison between groups 3 and 4, p = 0.073.

#### Patient Symptoms and Signs of HFpEF and Cardiac Function

Patient symptoms and signs of HFpEF, cardiac function, and the rate of cardiac events are shown in Table 4. Most patients had dyspnea on exertion. LV relaxation function, suggested by mean mitral e′, decreased in all groups. The mean mitral E/e′ ratio was higher in groups 2, 3, and 4 than in group 1. Group 1 exhibited the lowest rate of volume overload signs and symptoms, such as leg edema, neck vein dilatation, and pleural effusion. LV and RV function and morphology were preserved in group 1 compared with other groups. This group also exhibited a lower value of BNP (median 72 pg/ml). Group 2 demonstrated an intermediate rate of leg edema (40%). In group 2, LV and RV function and morphology were preserved compared with groups 3 and 4. Group 3 had a higher rate of volume overload signs and symptoms (the rate of leg edema and neck vein dilatation was 73 and 36%, respectively). Compared with groups 1 and 2, group 3 exhibited a larger LAVI, shorter deceleration time of mitral inflow (the rate of deceleration time ≤ 160 ms, 43%), more dilated right ventricle, lower TAPSE (the rate of TAPSE <17 mm, 45%), and larger inferior vena cava. Most patients in group 3 had less-distensible right ventricle. Group 4 demonstrated the highest rate of volume overload signs and symptoms. This group also had a higher SPAP and rate of less-distensible right ventricle, larger inferior vena cava, and the highest level of BNP (median BNP 288 pg/ml).

**Table 4:** Patient symptoms and sings of HF, cardiac function, and cardiac events according to stratification using k-means clustering.

	**Group 1 (*n* = 157)**	**Group 2 (*n* = 85)**	**Group 3 (*n* = 44)**	**Group 4 (*n* = 64)**	***p*-Value**
Symptoms and signs of HFpEF					
Dyspnea on exertion	154 (98)	84 (99)	41 (93)	58 (91)	0.018
Leg edema	16 (10)	34 (40)*	32 (73)^#$^	62 (97)^+!¶^	<0.001
Neck vein dilatation	6 (4)	10 (12)*	16 (36)^#$^	55 (86)^+!¶^	<0.001
Pleural effusion	1 (1)	7 (8)*	3 (7)	53 (83)^+!¶^	<0.001
Cardiac function					
Left heart function					
LAVI, ml/m^2^	35 (34–38)	39 (34–45)*	47 (39–58)^#$^	41 (35–51)^+¶^	<0.001
LVMI, g/m^2^	114 (97–136)	122 (103–150)	115 (101–142)	118 (102–141)	0.189
RWT > 0.42	91 (58)	55 (65)	25 (57)	34 (53)	0.535
LVEF, %	67 (61–74)	67 (61–72)	67 (61–71)	65 (58–73)	0.501
LVEDD, mm	49 (45–52)	46 (44–51)*	47 (45–52)	46 (43–50)	0.051
DT of mitral inflow, ms	217 (188–247)	223 (187–256)	167 (137–182)^#$^	187 (161–217)^+!¶^	<0.001
DT ≤ 160 ms	14 (9)	5 (6)	19 (43) ^#$^	16 (25) ^+!¶^	<0.001
Mean mitral e′, cm/s	7.7 (6.1–8.5)	7.3 (4.8–8.4)	7.4 (6.4–8.4)	7.9 (5.5–8.5)	0.145
Mean mitral E/e′ ratio	9 (8–12)	12 (9–16)*	13 (11–16)^#^	13 (10–16)^+^	<0.001
Mean mitral E/e′> 14	21 (13)	27 (32)*	20 (45)^#^	23 (36)^+^	<0.001
Right heart function					
RVOT, mm	26 (23–29)	26 (23–28)	28 (26–32)^#$^	26 (23–31)^¶^	<0.001
RV mid cavity diameter, mm	28 (25–33) (*n* = 143)	27 (24–30) (*n* = 74)	31 (29–34)^#$^ (*n* = 37)	32 (29–37)^+!^ (*n* = 53)	<0.001
RV basal diameter, mm	34 (29–39) (*n* = 143)	31 (29–34) (*n* = 74)	36 (30–41)^$^ (*n* = 37)	40 (32–43)^+!^ (*n* = 53)	<0.001
TAPSE, mm	21 (19–24)	20 (18–23)*	17 (15–20)^#$^	17 (16–19)^+!^	<0.001
TAPSE <17 mm	7 (4)	14 (16)*	20 (45)^#$^	29 (45)^+!^	<0.001
SPAP, mmHg	26 (21–31)	30 (25–38)*	31 (26–39)^#^	37 (29–42)^+!^	<0.001
SPAP > 35 mmHg	11 (7)	28 (33)*	15 (34)^#^	38 (59)^+!¶^	<0.001
Less-distensible right ventricle	18 (11)	19 (22)	41 (93)^#$^	57 (89)^+!^	<0.001
Inferior vena cava, mm	12 (10–15)	13 (10–15)	16 (13–20)^#$^	15 (13–20)^+!^	<0.001
Collapse with sniff <50%	1 (0)	11 (13)*	13 (30)^#$^	51 (80)^+!¶^	<0.001
Brain natriuretic peptide, pg/ml	72 (44–113)	207 (115–443)*	173 (117–319)^#^	288 (184–531)^+!¶^	<0.001
Cardiac events	8 (5)	20 (24)*	7 (16)^#^	45 (70)^+!¶^	<0.001

Data are the number of patients (%), median (interquartile range), or mean ± SD. RV, right ventricular; RWT, relative wall thickness. Other abbreviations are the same as those in Table 1. *, comparison between groups 1 and 2, p < 0.05; #, comparison between groups 1 and 3, p < 0.05; +, comparison between groups 1 and 4, p < 0.05; $, comparison between groups 2 and 3, p < 0.05; !, comparison between groups 2 and 4, p < 0.05; ¶, comparison between groups 3 and 4, p < 0.05.

#### Relationship Between Clinical Phenotypes and Patient Outcomes

**Figure 4:**
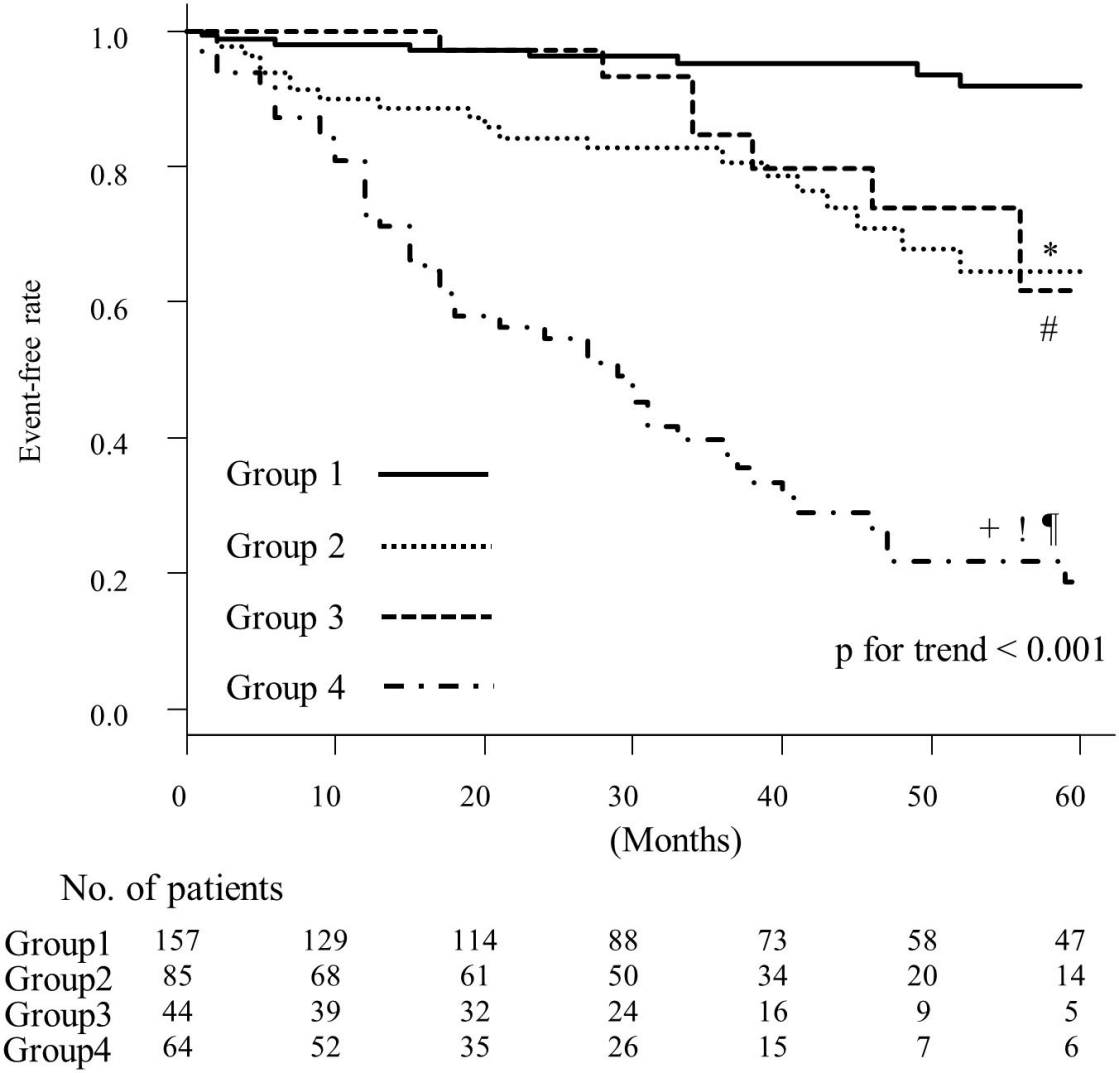
Kaplan–Meier curves for event-free rates according to the results of k-means clustering. *, comparison between groups 1 and 2, *p* < 0.05; #, comparison between groups 1 and 3, *p* < 0.05; +, comparison between groups 1 and 4, *p* < 0.05; !, comparison between groups 2 and 4, *p* < 0.05; ¶, comparison between groups 3 and 4, *p* < 0.05.

Cox proportional hazard analysis is shown in Table 5. Compared with group 1, group 4 exhibited the highest risk of cardiac events (hazard ratio [HR]: 19; 95% confidence interval [CI] 8.9–41); group 2 and 3 demonstrated similar cardiac event rates (group 2 HR: 5.1; 95% CI 2.2–12; group 3 HR: 3.7; 95%CI, 1.3–10). These results were almost the same in the age-adjusted model (Table 5). The Kaplan–Meier analysis of HFpEF stratified by k-means clustering is shown in Figure 4. The event-free rate was the lowest for patients in group 4 (*p* for trend < 0.001).

**Table 5:** Association of phenogroups with cardiac events on Cox proportional hazards analysis.

	**Group 1**	**Group 2**	**Group 3**	**Group 4**
Unadjusted HR (95% CI)				
Cardiac events	1.0	5.1 (2.2–12)^‡^	3.7 (1.3–10)^※^	19 (8.9–41)^‡^
Age adjusted model HR (95% CI)				
Cardiac events	1.0	5.6 (1.9–16)^†^	3.3 (1.1–9.7)^※^	15 (6.1–39)^‡^
Unadjusted HR (95% CI)				
Cardiac events	–	1.0	0.7 (0.3–1.7)	3.8 (2.2–6.4)^‡^
Age adjusted model HR (95% CI)				
Cardiac events	–	1.0	0.7 (0.3–18.8)	3.8 (2.2–6.4)^‡^
Unadjusted HR (95% CI)				
Cardiac events	–	–	1.0	5.6 (2.5–12)^‡^
Age adjusted model HR (95% CI)				
Cardiac events	–	–	1.0	4.6 (1.9–11)^‡^

*CI, confidence interval; HR, hazard ratio. ^※^p < 0.05; p < 0.01; ^‡^p < 0.001*.

### Validation of the K-Means Clustering

To validate our k-means clustering results, we retrospectively enrolled 133 outpatients with HFpEF. Clinical, laboratory, echocardiography, and jugular venous pulse characteristics of these 133 HFpEF participants are shown in Supplementary Table 2. The validation group had a higher systolic and mean blood pressure, and hemoglobin level, lower left ventricular mass index and left ventricular end-diastolic dimension, slower DT of mitral inflow, larger RV outflow tract, and higher TAPSE than the original group. However, there were no differences in age, AF, loop diuretics, eGFR, BNP, signs and symptoms of HFpEF, LAVI, mean mitral E/e′ ratio, SPAP, less-distensible right ventricle, or cardiac events. These indices for the original data exhibited a higher coefficient of principal component 1 (Supplementary Table 1), or important indices to distinguish between the clusters. The coefficient of principal component 1 for the validation data was almost the same as that for the original data (Supplementary Table 3). Group using validation data are shown in Supplementary Tables 4, 5. Significant differences among the groups in the validation cohort were almost the same as those in the original cohort. Group 2, 3, and 4 in the validation cohort, as in the original cohort, was associated with cardiac events independently of age, with hazard ratios comparable to those of the original cohort (Supplementary Table 6).

## Discussion

In the present study, patients with HFpEF were divided into four groups using k-means clustering. These groups had different etiologies and pathophysiologies. The event-free rates of cardiac events were significantly different among some groups. Using a cohort of 350 outpatients with documented HFpEF, and a validation cohort of 133 independent outpatients with HFpEF, we demonstrated the feasibility and validity of the k-means clustering technique for HFpEF.

Clustering is an unsupervised machine learning task that automatically divides the data into clusters, or groups of similar items and is used for knowledge discovery rather than prediction and it provides insight into the natural groupings found within data (30, 31). Thus, it is important that the clinical significance of dividing HFpEF is understood by physicians. K-means clustering is not as sophisticated as more modern clustering algorithms; however, it uses simple principles to find the nearest centroid for each point. Therefore, k-means clustering is an easy-to-understand clustering algorithm for physicians who are unfamiliar with machine learning, which may be a key advantage. Therefore, it may be easier for physicians to understand important functional features to specify HFpEF, such as renal function, AF, mean mitral E/e′ ratio, and RV systolic and diastolic function, which were used in our study, and the combination of complications leading to the poorer prognosis of patients with HFpEF.

### Group 1 (Younger Patients With Mild Symptoms and LV Relaxation Abnormality)

Our group 1 was the youngest and relatively higher BMI (the prevalence of obesity, 41%) and rate of prior coronary revascularization (35%). Generally, LV relaxation ability decreases with age; however, the mean mitral e′ of group 1 was the same as that of the other groups. Group 1 had LV relaxation abnormality which is the most common cardiac dysfunction of HFpEF. Obesity is associated with LVH and incipient LV dysfunction [5]. Ischemia can also influence the LV relaxation ability, even in the absence of overt ischemia, and it improves after coronary revascularization (35, 36). Thus, these comorbidities may be associated with poorer LV relaxation ability of group 1. LV relaxation abnormality may be associated with dyspnea on exertion through incomplete LV relaxation due to exercise-induced tachycardia (37). Considering these features, the pathophysiology of group 1 HFpEF resembles one of the previously reported phenotypes of HFpEF, exercise-induced diastolic dysfunction (38). Other cardiac functions and morphology were preserved in group 1, which may have been associated with the highest event-free rate among the groups.

### Group 2 (Older Patients With Renal Dysfunction)

Our group 2 was older and lower eGFR (the prevalence of CKD, 66%). LV and RV function and morphology were preserved, except for the rate of increase in LV filling pressure suggested by the mean mitral E/e′ ratio >14. The DT of mitral inflow in group 2 was slower compared with group 3 and 4, which suggested that LV filling depended more on slow filling (39). Although LV relaxation ability in group 2 seemed to be the same as that in group 1, the ratio of increase in LV filling pressure in group 2 was higher than that in group 1. The mechanisms of the increase in LV filling pressure may not be through advanced LV diastolic dysfunction, but instead through volume overload due to renal dysfunction in group 2. Excessive sodium retention increases the extracellular fluid volume in patients with renal failure (40). As the left ventricle is not a volume pump, but a pressure pump (16), excessive sodium retention due to renal dysfunction may cause an increase in the LV filling pressure under the condition of LV relaxation abnormality (41). Renal dysfunction may be also associated with the increase in the prevalence of volume overload signs in group 2. As an inverse relationship between renal function and adverse cardiovascular outcomes has been reported (42), the comorbidity of CKD may also have led to the poorer prognosis of HFpEF in group 2. As LV and RV function and morphology were preserved compared with groups 3 and 4, chronic renocardiac syndrome (cardiorenal syndrome type 4) was assumed to be the pathophysiology of group 2 in this study (41).

### Group 3 (AF and Advanced Biventricular Diastolic Dysfunction)

Most patients in group 3 had atrial fibrillation (95%), and advanced LV and RV dysfunction were more common than in groups 1 and 2. Among them, LAVI was the largest, DT of mitral inflow was shortest and the prevalence of less-distensible right ventricle was higher in group 3. HFpEF leads to AF via structural and functional remodeling of the left atrium. On the other hand, AF itself causes left atrial dilatation, impaired atrial function, and atrial fibrosis, AF may be a direct cause of HFpEF (43). Due to the elimination of atrial contraction, ventricular filling depends more on the rapid filling phase, suggested by the shorter DT of mitral inflow (39). LV diastolic function may be more impaired by AF because the rate of a higher mean mitral E/e′ ratio increased. Indeed, AF is also associated with LV myocardial fibrosis which in turn leads to LV diastolic dysfunction, and successful cardioversion is associated with improvement of LV filling (43). Recently, we demonstrated that AF is associated with a decrease in RV distensibility (17). The relationship between RV diastolic function and AF has not been fully established yet; however, a vicious cycle may be formed between the right side of the heart and AF, similar to the relationship between the left side of the heart and AF. Moreover, chronic high LV filling pressure impact RV diastolic function through ventricular interaction (44). LV and RV diastolic function in group 3 may deteriorate via these mechanisms. In particular, less-distensible right ventricle may play an important role in the higher prevalence of volume overload signs and cardiovascular event rates (18).

### Group 4 (Older Patients With RV Afterload Mismatch and Renal Dysfunction)

Our group 4 was characterized by older age, comorbidities, and cardiac dysfunction shared, similar to groups 2 and 3, e.g., AF, RV dysfunction, and renal dysfunction. Moreover, a higher SPAP (SPAP > 35 mmHg, 59%) was also a complication. Less-distensible right ventricle, higher SPAP, and renal dysfunction should be paid attention to in the pathophysiology of group 4. When RV preload reserves are lost, indicated by less-distensible right ventricle, the stroke volume decreases with increased RV afterload, resulting in RV afterload mismatch and further deterioration of the hemodynamics of HFpEF (18). RV failure negatively affects renal function through the increase in right atrial pressure, i.e., congestive kidney failure (5), which is related to a poorer prognosis of HFpEF (45). On the other hand, worsening renal function leads to sodium retention and may evoke volume overload under the condition in less-distensible right ventricle because the RV preload reserve is limited. Thus, a possible pathophysiological mechanism of group 4 is the formation of a vicious cycle between RV afterload mismatch and renal dysfunction. Volume overload signs and symptoms resisting loop diuretics and the poorest prognosis among the groups may have been caused by this vicious cycle.

### Comparison With HFpEF Groups Identified in Previous Studies

Some of these comorbidities and demographics to stratify patients with HFpEF were reported previously, however, our study had several differences. Compared with previous reports (9–13), our subjects were older (median age 77 years old) and, median age of our group 4 was 85 years. Using machine learning, a high age group similar to our group 4 has not been reported previously. The identified subgroups of HFpEF in this study had more hemodynamic concepts than those in previous reports because LV and RV function, especially RV distensibility were taught in machine learning. Echocardiography is excellent for the assessment of cardiac function, but in comparison with LV diastolic function, there is a lack of guidance for the assessment and quantification of RV diastolic function (15). The examination of tricuspid inflow was recommended for the assessment of RV diastolic function (26); however, the echocardiographic assessment of RV function is often difficult due to the complex RV anatomy and these measures do not typically form part of a standard clinical echocardiographic study (15, 46). Indeed, RV diastolic function assessed using tricuspid inflow were not taught in previous reports (9–13). To overcome this problem, we paid attention to the jugular venous pulse. This method may be forgotten in RV assessment, but the waveform pattern can reflect the condition of the right ventricle (22, 23). Indeed, we previously reported that the combination of a high RV systolic pressure and less-distensible right ventricle, a situation in which RV afterload mismatch is easily evoked, exhibited the poorest outcomes in HFpEF (18) and that beta-blockers may be useful for the patients with HFpEF and preserved RV distensibility (20). The assessment of jugular venous pulse waveform is useful for the stratification of HFpEF. We hypothesize that the complications of RV afterload mismatch and renal dysfunction are associated with the poorest outcomes of HFpEF. Thus, to our best knowledge, this is the first study in which RV distensibility assessed by jugular venous pulse was utilized to divide HFpEF by machine learning and we clarified a new phenotype of older age for HFpEF using k-means clustering.

### Clinical Implication in the Pathophysiology of Group 4

The relationship between RV afterload mismatch and renal dysfunction is troublesome when deciding therapeutic strategies for patients in group 4. Only diuretics can improve volume overload and possibly the hemodynamics in HFpEF. Diuretics may improve congestive kidney disease, but their excessive use reduces the RV filling pressure, which reduces the stroke volume and may result in prerenal failure (22). Heart rate reduction may exert untoward action in patients with HFpEF and RV afterload mismatch because cardiac output depends more on heart rate. Our study also suggested that progression to cardio renal syndrome associated with RV afterload mismatch should be prevented by appropriate treatments.

### Study Limitations

Several methodological limitations must be considered. First, this was a retrospective study that was conducted at a single center and performed on consecutive patients with matching eligibility criteria. As we required satisfactory imaging of echocardiography and jugular venous pulse, some patients, such as markedly obese patients with limited windows or fatty neck, may have been underrepresented. Moreover, patients with tachycardia may also have been excluded because of difficulty in separating the E and A waves in the mitral inflow or the “X” and “Y” descent of the jugular venous pulse, as described previously (17, 18, 20). Second, it is well-known that wild-type transthyretin amyloidosis is an underdiagnosed cause of HFpEF (47). If patients had a dominant Y descent in the jugular venous pulse waveform, constrictive pericarditis and/or cardiac amyloidosis were suspected. These diseases were examined as in our previous study (17, 18, 20). However, our screening examination, such as echocardiography, may have been insufficient to detect ATTRwt. Other screening examinations with a higher sensitivity, such as scintigraphy, is needed (47). Thus, early stages of amyloidosis may have been included in the present study. Third, k-means clustering is not as sophisticated as more modern clustering algorithms. As it uses an element of random chance, it is not guaranteed to find the optimal set of clusters and requires a reasonable guess as to how many clusters naturally exist in the data. Stratified data by k-means clustering often include subjectivity of the researcher even if a technique, such as Hartigan's rule, to find the optimal k is applied (31). Using k-means clustering, categorical data need to be converted to numerical data. Clustering methods also have a risk of overlap of characteristics. Although we performed validation study to confirm our results, modern clustering algorithms that can analyze both categorical and numerical data may be more suitable for more precise stratification. Clustering methods have advanced since the inception of k-means and modern clustering algorithms may be superior; however, this does not mean that k-means is obsolete. K-means clustering is still used widely because of its simple principals, high flexibility, and satisfactory performance in many cases. The performance of a clustering algorithm depends on both the quality of the clusters themselves and what is done with the information (31). Moreover, as all learning algorithms are only as good as the input data, the features taught to the machine learning algorithm are also important. Indeed, by learning RV diastolic function, we clarified a new phenotype of older age for HFpEF. Fourth, the criteria for HFpEF are updated once every few years and patients meeting the latest criteria were enrolled retrospectively in this study; therefore, many patients were excluded from the original sample, which may have caused selection bias. Lastly, this retrospective study was unable to establish a causal relationship. However, our results may be useful for the management of elderly patients with HFpEF because the prevalence of HFpEF will increase with age. Thus, further prospective clinical studies are warranted to confirm our results.

## Conclusion

K-means clustering divided HFpEF into four groups. Older patients with HFpEF may suffer from complication of RV afterload mismatch and renal dysfunction. Our results may be useful for stratified medicine for HFpEF.

## Data Availability Statement

The raw data supporting the conclusions of this article will be made available by the authors, without undue reservation.

## Ethics Statement

The studies involving human participants were reviewed and approved by Imizu Municipal Hospital Ethics/Clinical Trial Review Committee. The patients/participants provided their written informed consent to participate in this study.

## Author Contributions

DH and HA worked on the conception, methodology, and formal analysis. Data collection was performed by DH, TN, and JT. DH wrote the manuscript. All authors approved the final version of the manuscript.

## Conflict of Interest

HA received a research grant from Sun Medical Technology Research Corp., Sumitomo Riko Company Limited., Century Medical, Inc., Teijin Pharma Limited., Nipro Corporation., Medtronic Japan Co, Ltd. The remaining authors declare that the research was conducted in the absence of any commercial or financial relationships that could be construed as a potential conflict of interest.
