# Supplementary material for: Different Pathophysiology and Outcomes of Heart Failure With Preserved Ejection Fraction Stratified by K-Means Clustering
Source: Front Cardiovasc Med. 2020 Nov 30;7:607760. doi: 10.3389/fcvm.2020.607760 (PMC7734143; doi:10.3389/fcvm.2020.607760)
Supplement: Supplementary file 1 [file Table_1.DOCX]

Supplementary Table 1 Coefficient of each feature to create the axes of PC 1 and 2

|  | PC1 | PC2 |
| --- | --- | --- |
| Age | 0.237 | -0.274 |
| Male | -0.081 | 0.334 |
| Body mass index | -0.125 | 0.138 |
| Heart rate | 0.086 | 0.002 |
| Systolic blood pressure | 0.017 | -0.077 |
| Diastolic blood pressure | -0.007 | -0.069 |
| Mean blood pressure | 0.002 | -0.083 |
| Underlying disorders |  |  |
| Hypertension | 0.045 | -0.083 |
| Diabetes mellitus | -0.009 | 0.048 |
| Hyperlipidemia | -0.140 | 0.001 |
| Chronic obstructive pulmonary disease | 0.075 | 0.103 |
| Prior coronary revascularization | -0.093 | 0.017 |
| Atrial fibrillation | 0.221 | 0.319 |
| Medications |  |  |
| ACEI/ARB | 0.038 | -0.065 |
| Beta-blockers | -0.046 | 0.101 |
| Calcium channel blockers | 0.011 | -0.071 |
| Loop diuretics | 0.270 | -0.026 |
| eGFR | -0.162 | 0.160 |
| Hemoglobin | -0.225 | 0.192 |
| Brain natriuretic peptide | 0.207 | -0.102 |
| Symptoms and signs of HFpEF |  |  |
| Dyspnea on exertion | -0.065 | -0.010 |
| Leg edema | 0.296 | 0.075 |
| Neck vein dilatation | 0.281 | 0.086 |
| Pleural effusion | 0.273 | -0.002 |
| Cardiac function |  |  |
| Left heart |  |  |
| LAVI | 0.166 | 0.199 |
| LVMI | 0.034 | 0.215 |
| LVEF | -0.023 | -0.230 |
| LVEDD | -0.054 | 0.361 |
| DT of mitral inflow | -0.079 | -0.210 |
| Mean mitral | -0.047 | 0.130 |
| Mean mitral E/e’ ratio | 0.154 | -0.050 |
| Right heart |  |  |
| RVOT | 0.037 | 0.218 |
| TAPSE | -0.202 | -0.062 |
| SPAP | 0.227 | 0.091 |
| Less-distensible right ventricle | 0.261 | 0.135 |
| Inferior vena cava | 0.187 | 0.273 |
| Cardiac events | 0.247 | 0.012 |

Data are the coefficient of each feature to create the axes of principle components 1 and 2. ACEI/ARB, angiotensin-converting enzyme inhibitors/angiotensin-receptor blockers; DT, deceleration time; eGFR, estimated glomerular filtration rate; HFpEF, heart failure with preserved ejection fraction; LAVI, left atrial volume index; LVEDD, left ventricular end-diastolic dimension; LVEF, left ventricular ejection fraction; LVMI, left ventricular mass index; PC, principle component; RVOT, right ventricular outflow tract; SPAP, systolic pressure arterial pressure; TAPSE, tricuspid annular plane systolic excursion.
