# Supplementary material for: Different Pathophysiology and Outcomes of Heart Failure With Preserved Ejection Fraction Stratified by K-Means Clustering
Source: Front Cardiovasc Med. 2020 Nov 30;7:607760. doi: 10.3389/fcvm.2020.607760 (PMC7734143; doi:10.3389/fcvm.2020.607760)
Supplement: Supplementary file 2 [file Table_2.DOCX]

Supplementary Table 2 Patient characteristics: Original vs Validation data

|  | Original (n=350) | Validation (n=133) | p-value |
| --- | --- | --- | --- |
| Age, years | 77 (69-83) | 74 (67-83) | 0.127 |
| Male | 159 (45) | 69 (52) | 0.222 |
| Body mass index, kg/m^2^ | 24.0±3.7 | 23.9±4.0 | 0.716 |
| Heart rate, bpm | 68 (62-76) | 66 (58-75) | 0.071 |
| Systolic blood pressure, mmHg | 121 (109-133) | 126 (118-135) | <0.001 |
| Diastolic blood pressure, mmHg | 69 (59-82) | 72 (65-79) | 0.302 |
| Mean blood pressure, mmHg | 87 (79-95) | 89 (83-96) | 0.005 |
| Underlying disorders |  |  |  |
| Hypertension | 310 (89) | 125 (94) | 0.089 |
| Diabetes mellitus | 71 (20) | 32 (24) | 0.385 |
| Hyperlipidemia | 116 (33) | 44 (33) | 1 |
| COPD | 34 (10) | 13 (10) | 1 |
| Prior coronary revascularization | 86 (25) | 36 (27) | 0.561 |
| Atrial fibrillation | 82 (23) | 29 (22) | 0.809 |
| Medications |  |  |  |
| ACEI/ARB | 250 (71) | 92 (69) | 0.655 |
| Beta-blockers | 187 (53) | 66 (50) | 0.476 |
| Calcium channel blockers | 188 (54) | 63 (47) | 0.223 |
| Loop diuretics | 158 (45) | 49 (37) | 0.122 |
| eGFR, ml/min/1.73m^2^ | 62 (49-76) | 64 (51-76) | 0.217 |
| Hemoglobin, g/dl | 12.5 (11.1-13.6) | 13.2 (11.7-14.2) | <0.001 |
| Brain natriuretic peptide, pg/ml | 131 (69-267) | 117 (73-177) | 0.263 |
| Symptoms and signs of HFpEF |  |  |  |
| Dyspnea on exertion | 337 (96) | 132 (99) | 0.126 |
| Leg edema | 144 (41) | 48 (36) | 0.349 |
| Neck vein dilatation | 87 (25) | 23 (17) | 0.089 |
| Pleural effusion | 64 (18) | 17 (13) | 0.173 |
| Cardiac function |  |  |  |
| Left heart |  |  |  |
| LAVI, ml/m^2^ | 38 (34-44) | 38 (35-47) | 0.313 |
| LVMI, g/m^2^ | 116 (100-141) | 108 (95-122) | 0.003 |
| LVEF, % | 67 (60-73) | 68 (61-73) | 0.833 |
| LVEDD, mm | 48 (44-52) | 47 (43-50) | 0.012 |
| DT of mitral inflow | 208 (173-241) | 221 (187-252) | 0.022 |
| Mean mitral e’, cm/s | 7.6 (5.8- 8.5) | 7.1 (6.1-7.8) | 0.287 |
| Mean mitral E/e’ ratio | 10.6 (8.4-14.2) | 10.8 (8.0-14.1) | 0.694 |
| Right heart |  |  |  |
| RVOT, mm | 26 (23-30) | 28 (26-31) | <0.001 |
| TAPSE, mm | 20 (18-23) | 21 (18-25) | 0.008 |
| SPAP, mmHg | 29 (23-36) | 28 (24-31) | 0.072 |
| Less-distensible right ventricle | 135 (39) | 40 (30) | 0.091 |
| Inferior vena cava, mm | 13 (11-16) | 13 (10-15) | 0.056 |
| Cardiac events | 80 (23) | 36 (27) | 0.342 |

Data are the number of patients (%), median (interquartile range), or mean ± SD. Abbreviations are the same as those in Supplementary Table 1.
