# Supplementary material for: Different Pathophysiology and Outcomes of Heart Failure With Preserved Ejection Fraction Stratified by K-Means Clustering
Source: Front Cardiovasc Med. 2020 Nov 30;7:607760. doi: 10.3389/fcvm.2020.607760 (PMC7734143; doi:10.3389/fcvm.2020.607760)
Supplement: Supplementary file 3 [file Table_3.DOCX]

Supplementary Table 3 Coefficient of each feature to create the axes of PC 1 and 2 for validation data

|  | PC1 | PC2 |
| --- | --- | --- |
| Age | 0.219 | 0.039 |
| Male | -0.115 | -0.174 |
| Body mass index | -0.133 | -0.114 |
| Heart rate | 0.107 | 0.334 |
| Systolic blood pressure | 0.061 | 0.172 |
| Diastolic blood pressure | -0.049 | 0.284 |
| Mean blood pressure | -0.012 | 0.309 |
| Underlying disorders |  |  |
| Hypertension | -0.011 | -0.082 |
| Diabetes mellitus | -0.011 | -0.170 |
| Hyperlipidemia | -0.056 | -0.168 |
| Chronic obstructive pulmonary disease | 0.049 | -0.176 |
| Prior coronary revascularization | -0.060 | -0.230 |
| Atrial fibrillation | 0.235 | 0.001 |
| Medications |  |  |
| ACEI/ARB | 0.111 | -0.113 |
| Beta-blockers | 0.055 | -0.238 |
| Calcium channel blockers | 0.002 | 0.037 |
| Loop diuretics | 0.220 | 0.125 |
| eGFR | -0.194 | -0.056 |
| Hemoglobin | -0.230 | -0.009 |
| Brain natriuretic peptide | 0.273 | -0.057 |
| Symptoms and signs of HFpEF |  |  |
| Dyspnea on exertion | -0.098 | -0.074 |
| Leg edema | 0.285 | -0.002 |
| Neck vein dilatation | 0.292 | -0.101 |
| Pleural effusion | 0.274 | -0.028 |
| Cardiac function |  |  |
| Left heart |  |  |
| LAVI | 0.204 | -0.105 |
| LVMI | 0.084 | -0.218 |
| LVEF | -0.013 | 0.114 |
| LVEDD | -0.113 | -0.366 |
| DT of mitral inflow | -0.042 | 0.033 |
| Mean mitral | -0.042 | 0.033 |
| Mean mitral E/e’ ratio | 0.197 | -0.045 |
| Right heart |  |  |
| RVOT | -0.085 | -0.146 |
| TAPSE | -0.146 | -0.084 |
| SPAP | 0.239 | -0.054 |
| Less-distensible right ventricle | 0.261 | 0.135 |
| Inferior vena cava | 0.075 | -0.076 |
| Cardiac events | 0.269 | -0.136 |

Data are the coefficient of each feature to create the axes of principle components 1 and 2. Abbreviations are the same as those in Supplementary Table 1.
