# Supplementary material for: Different Pathophysiology and Outcomes of Heart Failure With Preserved Ejection Fraction Stratified by K-Means Clustering
Source: Front Cardiovasc Med. 2020 Nov 30;7:607760. doi: 10.3389/fcvm.2020.607760 (PMC7734143; doi:10.3389/fcvm.2020.607760)
Supplement: Supplementary file 4 [file Table_4.DOCX]

Supplementary Table 4 Patient characteristics and comorbidities according to stratification using k-means clustering for validation data

|  | Group 1  (n = 64) | Group 2  (n = 30) | Group 3  (n = 21) | Group 4  (n = 18) | p-value |
| --- | --- | --- | --- | --- | --- |
| Age, years | 70 (60-76) | 82 (72-84) | 79 (71-86) | 85 (76-89) | <0.001 |
| Male | 46 (72) | 6 (20) | 10 (48) | 7 (39) | 0.001 |
| BMI, kg/m^2^ | 24 (22-26) | 24 (20-27) | 23 (21-25) | 21 (19-23) | 0.019 |
| Heart rate, bpm | 62 (57-69) | 69 (59-75) | 72 (61-76) | 73 (68-83) | 0.007 |
| Systolic blood pressure, mmHg | 125 (120-132) | 127 (118-136) | 128 (116-140) | 129 (119-141) | 0.604 |
| Diastolic blood pressure, mmHg | 72 (66-77) | 71 (61-82) | 72 (68-82) | 69 (60-78) | 0.472 |
| Mean blood pressure, mmHg | 90 (84-95) | 88 (82-97) | 90 (85-100) | 89 (82-96) | 0.713 |
| Underlying disorders |  |  |  |  |  |
| Hypertension | 56 (92) | 29 (97) | 20 (95) | 17 (94) | 0.947 |
| Diabetes mellitus | 16 (25) | 5 (17) | 7 (33) | 4 (22) | 0.595 |
| Hyperlipidemia | 28 (44) | 5 (17) | 8 (38) | 3 (17) | 0.024 |
| COPD | 6 (9) | 1 (3) | 2 (10) | 4 (22) | 0.213 |
| Prior coronary revascularization | 24 (38) | 7 (23) | 4 (19) | 1 (6) | 0.033 |
| Atrial fibrillation | 1 (2) | 0 (0) | 19 (90) | 9 (50) | <0.001 |
| Medications |  |  |  |  |  |
| ACEI/ARB | 40 (63) | 19 (63) | 18 (86) | 15 (83) | 0.109 |
| Beta-blockers | 30 (47) | 12 (40) | 14 (67) | 10 (56) | 0.268 |
| Calcium channel blockers | 28 (44) | 16 (53) | 10 (48) | 9 (50) | 0.856 |
| Loop diuretics | 5 (8) | 15 (50) | 15 (71) | 18 (78) | <0.001 |
| eGFR, ml/min/1.73m^2^ | 73 (63-85) | 52 (47-63) | 60 (49-71) | 47 (32-64) | <0.001 |
| Hemoglobin, mg/dl | 14±1 | 12±2 | 13±2 | 11±2 | <0.001 |

Data are the number of patients (%), median (interquartile range), or mean ± SD. Abbreviations are the same as those in Supplementary Table 1.
