# Supplementary material for: Different Pathophysiology and Outcomes of Heart Failure With Preserved Ejection Fraction Stratified by K-Means Clustering
Source: Front Cardiovasc Med. 2020 Nov 30;7:607760. doi: 10.3389/fcvm.2020.607760 (PMC7734143; doi:10.3389/fcvm.2020.607760)
Supplement: Supplementary file 5 [file Table_5.DOCX]

Supplementary Table 5 Patient symptoms and signs of HF, cardiac function and cardiac events according to stratification using k-means clustering for validation data

|  | Group 1  (n = 64) | Group 2  (n = 30) | Group 3  (n = 21) | Group 4  (n = 18) | p-value |
| --- | --- | --- | --- | --- | --- |
| Symptoms and signs of HFpEF |  |  |  |  |  |
| Dyspnea on exertion | 65 (100) | 30 (100) | 21 (100) | 17 (94) | 0.241 |
| Leg edema | 4 (6) | 11 (37) | 15 (71) | 18 (100) | <0.001 |
| Neck vein dilatation | 0 (0) | 0 (0) | 5 (24) | 18 (100) | <0.001 |
| Pleural effusion | 0 (0) | 0 (0) | 1 (5) | 16 (89) | <0.001 |
| Cardiac function |  |  |  |  |  |
| Left heart function |  |  |  |  |  |
| LAVI, ml/m^2^ | 36 (34-39) | 38 (35-40) | 55 (48-67) | 47 (41-52) | <0.001 |
| LVMI, g/m^2^ | 104 (93-120) | 108 (94-118) | 115 (105-131) | 115 (101-135) | 0.119 |
| LVEF, % | 68 (62-71) | 68 (62-72) | 69 (61-73) | 65 (56-69) | 0.384 |
| LVEDD, mm | 49 (45-52) | 43 (40-46) | 47 (41-51) | 43 (41-47) | <0.001 |
| DT of mitral inflow, ms | 222 (195-251) | 241 (206-279) | 184 (158-227) | 199 (173-239) | 0.002 |
| Mean mitral e’, cm/s | 7.1 (5.9-8.4) | 6.9 (5.5-7.2) | 7.3 (6.5-9.4) | 6.9 (6.5-7.2) | 0.102 |
| Mean mitral E/e’ ratio | 9 (7-11) | 13 (9-16) | 13 (9-16) | 14 (12-19) | <0.001 |
| Right heart function |  |  |  |  |  |
| RVOT, mm | 28 (27-31) | 27 (24-30) | 30 (27-31) | 26 (24-29) | 0.018 |
| TAPSE, mm | 23 (19-26) | 22 (18-24) | 19 (15-22) | 18 (16-20) | <0.001 |
| SPAP, mmHg | 25 (23-28) | 28 (24-31) | 30 (28-32) | 36 (34-36) | <0.001 |
| Less-distensible right ventricle | 1 (2) | 3 (10) | 21 (100) | 15 (83) | <0.001 |
| Inferior vena cava, mm | 13 (10-15) | 12 (9-14) | 14 (13-15) | 14 (11-17) | 0.021 |
| Brain natriuretic peptide, pg/ml | 74 (62-96) | 131 (120-186) | 211 (120-247) | 264 (165-408) | <0.001 |
| Cardiac events | 5 (8) | 9 (30) | 6 (29) | 16 (89) | <0.001 |

Data are the number of patients (%), median (interquartile range) or mean ± SD. Abbreviations are the same as those in Supplementary Table 1.
