# Supplementary material for: Different Pathophysiology and Outcomes of Heart Failure With Preserved Ejection Fraction Stratified by K-Means Clustering
Source: Front Cardiovasc Med. 2020 Nov 30;7:607760. doi: 10.3389/fcvm.2020.607760 (PMC7734143; doi:10.3389/fcvm.2020.607760)
Supplement: Supplementary file 6 [file Table_6.DOCX]

Supplementary Table 6 Association of phenogroups groups with cardiac events on Cox proportional hazards analysis for validation data

|  | Group 1 | Group 2 | Group 3 | Group 4 |
| --- | --- | --- | --- | --- |
| Unadjusted HR (95% CI) |  |  |  |  |
| Cardiac events | 1.0 | 3.8 (1.3-12)^※^ | 3.3 (1.0-11)^※^ | 20 (7.1-56)‡ |
| Age adjusted model HR (95% CI) |  |  |  |  |
| Cardiac events | 1.0 | 4.6 (1.2-17)^※^ | 3.7 (1.1-13)^※^ | 17 (5.2-53)‡ |
| Unadjusted HR (95% CI) |  |  |  |  |
| Cardiac events | - | 1.0 | 0.9 (0.3-2.5) | 5.1 (2.2-12)‡ |
| Age adjusted model HR (95% CI) |  |  |  |  |
| Cardiac events | - | 1.0 | 0.9 (0.3-2.6) | 4.6 (2.0-1)‡ |
| Unadjusted HR (95% CI) |  |  |  |  |
| Cardiac events | - | - | 1.0 | 5.6 (2.1-15)‡ |
| Age adjusted model HR (95% CI) |  |  |  |  |
| Cardiac events | - | - | 1.0 | 4.9 (1.9-13)^†^ |

CI, confidence interval; HR, hazard ratio. ※p<0.05; †p<0.01; ‡p<0.001
